# Viral suppression and self-reported ART adherence after 3 years of universal testing and treatment in the HPTN 071 (PopART) community-randomised trial in Zambia and South Africa: a cross-sectional analysis

**DOI:** 10.1016/S2352-3018(22)00237-5

**Published:** 2022-11-01

**Authors:** David Macleod, Kwame Shanaube, Timothy Skalland, Mohammed Limbada, Nomtha Mandla, Justin Bwalya, Ab Schaap, Blia Yang, Deborah Donnell, Estelle Piwowar-Manning, Susan H Eshleman, Graeme Hoddinott, Virginia Bond, Ayana Moore, Sam Griffith, Peter Bock, Helen Ayles, Sarah Fidler, Richard Hayes, Sian Floyd, HPTN 071 (PopART) Study Team

**Affiliations:** a International Statistics and Epidemiology Group, Department of Infectious Disease Epidemiology, London School of Hygiene & Tropical Medicine, London, UK; b Global Health and Development Department, Faculty of Public Health and Policy, London School of Hygiene & Tropical Medicine, London, UK; c Zambart, School of Public Health, University of Zambia, Lusaka, Zambia; d HPTN Statistical and Data Management Centre, Seattle, WA, USA; e Desmond Tutu TB Centre, Department of Paediatrics and Child Health, Stellenbosch University, Cape Town, South Africa; f Department of Pathology, Johns Hopkins University School of Medicine, Baltimore, MD, USA; g FHI 360, HIV Prevention Trials Network, Durham, NC, USA; h Department of Infectious Disease, Faculty of Medicine, Imperial College London, London, UK

## Abstract

**Background:**

In 2014, UNAIDS set the target that 90% of individuals on antiretroviral therapy (ART) be virally suppressed. Here, we use data from the HPTN 071 (PopART) trial to report whether the introduction of universal testing and treatment has affected viral suppression or treatment adherence among individuals who self-reported they were taking ART, and identify risk factors for these outcomes.

**Methods:**

This was a cross-sectional study nested within the randomly selected population cohort of the PopART trial. The trial took place in 21 communities in Zambia and South Africa. Analyses included 3570 HIV-positive participants who were seen at the second follow-up visit in 2016–17 and who self-reported that they were currently taking ART. Viral suppression was defined as HIV RNA of less than 400 copies per mL from a blood sample collected during the cohort visit, and ART adherence was measured using self-reporting (reported as no missed pills in last 7 days). Prevalences of these outcomes were compared across three trial arms using a two-stage approach suitable for clustered data. Each arm consisted of seven communities, with one arm receiving a combination HIV prevention package including immediate ART initiation, one receiving a combination HIV prevention package excluding immediate ART initiation and one arm receving standard of care. Risk factors for each of the outcomes were assessed using logistic regression.

**Findings:**

Among the 3570 participants who self-reported that they were currently on ART, 416 (11·7%) of 3554 were not virally suppressed (16 were missing viral suppression status) and 345 (9·7%) of 3566 reported being non-adherent to ART (four were missing adherence status). The proportion not virally suppressed was higher in communities in South Africa (195 [16·4%] of 1191) than in Zambia (221 [9·4%] of 2363). There was no evidence that the prevalence of the outcomes differed between trial arms. There was evidence that men, younger individuals, individuals who reported participating in harmful alcohol use, and those who reported internalised stigma were more likely to be non-adherent, and not virally suppressed.

**Interpretation:**

The results assuaged concerns that early ART initiation in a universal testing and treatment setting could lead to reduced adherence and viral suppression.

**Funding:**

US National Institute of Allergy and Infectious Diseases (which is a part of the National Institutes of Health), the International Initiative for Impact Evaluation with support from the Bill & Melinda Gates Foundation, US President's Emergency Plan for AIDS Relief, and Medical Research Council UK.

## Introduction

In 2015, WHO revised guidelines based on randomised trial findings that recommended all people living with HIV start antiretroviral therapy (ART) irrespective of CD4 cell count or disease stage.^1^, ^2^, ^3^ This recommendation provided a strong rationale for the UNAIDS 90–90–90 targets for 2020 (ie, that 90% of people living with HIV would know their HIV status, 90% of those aware of their HIV-positive status would be receiving ART, and 90% of those on ART would be virally suppressed) and 95–95–95 targets for 2030.^4^, ^5^ To meet these goals, many countries in sub-Saharan Africa have scaled up ART programmes.

South Africa and Zambia are among the countries in sub-Saharan Africa that are most affected by HIV, with approximately 7·5 million people living with HIV in South Africa and 1·2 million people living with HIV in Zambia. By 2019, more than 70% of people living with HIV in these countries were on ART,^6^ but achieving lifelong retention in care and ART adherence is a big challenge in resource-limited settings.^7^ Low adherence can lead to suboptimal viral suppression with associated increases in HIV drug resistance, HIV transmission, and morbidity and mortality.^8^, ^9^ High adherence to ART requires retention in care, and is facilitated by accurate, consistent monitoring, which can be challenging in resource-limited settings.^10^, ^11^ A meta-analysis of 27 early studies of the use of ART in sub-Saharan Africa in 2002–06 showed fairly high rates of adherence in people living with HIV to ART with an estimated 77% achieving adequate adherence levels.^12^ However, with the initiation of ART earlier in infection due to the 2015 guidelines change, there was a concern that people living with HIV who had not experienced HIV-related ill health might be less likely to stay in care and adhere to treatment.^13^

**Research in context**

**Evidence before this study**

We searched PubMed on July 1, 2021, using the search terms “HIV” and “universal test and treat” or “universal testing and treatment” and either “viral suppression” or “adherence”, published in English only. Among the results, there was one relevant study identified, which was nested within the ANRS 12249 TasP trial in South Africa, which found no evidence of an association between suboptimal adherence to antiretroviral therapy (ART) and CD4 cell count at initiation in the first year of ART. Through using two different measures of adherence (a visual analogue scale and pill counts), the estimated odds ratios (ORs) were 1·00 (95% CI 0·95–1·05) and 1·03 (0·99–1·07) for an increase in CD4 count of 100 cells per μL.

**Added value of this study**

We had data from a large, community-randomised trial of universal testing and treatment in Zambia and the Western Cape of South Africa, allowing us to obtain estimates of both viral suppression and ART adherence among a random sample of people living with HIV who were on ART and retention on ART among people living with HIV who had ever taken ART. The data allowed us to make a randomised comparison of these outcomes between communities receiving a universal testing and treatment intervention and control communities. A strength of this study was that it was a very large sample of people living with HIV, randomly selected from a range of communities across two countries, which helps with generalisability and reliability of findings.

**Implications of all the available evidence**

ART adherence and viral suppression among people living with HIV who are on ART can be high in the context of universal testing and treatment, and we found no evidence to suggest that these outcomes were different among residents of universal testing and treatment communities compared with those in the control communities. This finding coupled with the earlier study provides reassurance that individuals initiating ART sooner are no less likely to adhere to treatment in the early years of receiving ART.

Universal testing and treatment combines the community-wide offer of HIV testing and counselling with ART for all people living with HIV. Four community- randomised trials have collectively provided evidence that universal testing with active linkage to care and access to ART can increase population-level viral suppression in sub-Saharan Africa, ultimately reducing HIV incidence.^14^ The HPTN 071 (PopART) trial^15^ was the largest of these trials taking place in 21 communities (total population of approximately 1 million people) in South Africa and Zambia. This trial provided an opportunity to evaluate ART adherence among people living with HIV in communities receiving universal testing and treatment and control communities using self-reported ART adherence and also laboratory-measured viral suppression data. The intervention could have either enhanced ART adherence due to the additional support and HIV messaging delivered throughout the communities, or could have reduced adherence due to a proportion of ART initiations being among clinically asymptomatic individuals who might feel less motivated to continue ART.

In this study, we analyse cross-sectional data obtained from the HPTN 071 (PopART) trial between May, 2016, and May, 2017, following 3 years of delivery of a universal testing and treatment intervention. We aimed to assess whether there were differences in viral suppression and self-reported ART adherence between the control and intervention communities among individuals who self-reported that they were taking ART, and aimed to identify risk factors associated with these outcomes.

## Methods

### Study design, participants, and procedures

This cross-sectional analysis was nested within the randomly selected population cohort of the HPTN 071 (PopART) trial. The three-arm community-randomised trial was conducted in 12 Zambian and nine South African communities from Nov 28, 2013, to Aug 2, 2018. Groups of three communities (so-called triplets) were matched on geographical area and HIV prevalence and then randomly assigned to each of the three study arms. Residents in arm A and arm B communities were offered a combination HIV prevention package, with those in arm A additionally offered immediate ART initiation regardless of CD4 count. Residents in arm C received standard of care services.

The HIV prevention package included HIV counselling, HIV rapid testing, linkage to care, support for retention in care and ART adherence, and was implemented in the two intervention arms (A and B) from December, 2013, to December, 2017, through annual household visits by trial-employed community HIV care providers (CHiPs). To support ART adherence, people living with HIV received follow-up visits in the weeks following the annual visit to monitor adherence and check whether it was proving to be difficult. Available support included: developing a plan with the participant for taking the drugs that was easy to follow and also worked alongside their daily activities; providing education on the goals of therapy, side-effects, and possible results of non-adherence; and identifying trusted treatment supporters with whom they were comfortable and encouraging those supporters to attend counselling sessions and clinic visits. CHiPs also collaborated with clinics to identify individuals who had missed appointments, following which they made additional home visits. There was also a focus on linking patients to existing adherence support for treating and managing side-effects, to services for treating depression or substance abuse, to adherence clubs in which these were available, and to SMS reminder services.

While delivering the intervention, CHiPs collected data relevant to delivery of their services (CHiP data). In arm A, participants identified as HIV-positive were offered ART immediately, irrespective of CD4 cell count. In arms B and C, ART was provided according to national guidelines, which were initially given when CD4 counts fell below 350 cells per μL. This threshold was increased during 2014 to a CD4 count of up to 500 cells per μL, then during 2016 to initiation regardless of CD4 cell count.

HIV incidence was assessed in a population cohort of 48 301 participants obtained by randomly sampling households and then randomly selecting one individual aged 18–44 years from each household. After enrolment, participants were revisited at 12, 24, and 36 months. At each visit, a structured questionnaire was completed with a research assistant, who collected demographic, socioeconomic, and behavioural data (population cohort data). Participants were asked about their HIV status; if they reported they were HIV-positive, they were asked whether they were currently, or had ever been, on ART, and if currently on ART, whether they had missed pills in the last 7 days. After the interview, a blood sample was taken by a research nurse for laboratory-based assessments.

Laboratory-based HIV tests that detect both antigens and antibodies were performed in central laboratories in South Africa and Zambia, with additional testing for quality control at the HPTN Laboratory Centre in Baltimore, USA.^16^ At the 24-month visit, viral load measurements were also obtained for all HIV-positive participants. HIV viral load testing was performed using the Abbott RealTime HIV-1 Viral Load Assay (Abbott Molecular, Des Plaines, USA). A validated dilution method was used for testing (limit of quantification: 400 copies of HIV RNA per mL).

Ethical approval was granted by the University of Zambia, Stellenbosch University, and London School of Hygiene & Tropical Medicine. Population cohort participants provided informed written consent before enrolment. Intervention participants provided verbal consent to participate and written informed consent for HIV testing. Individuals who initiated ART outside of national guidelines provided written informed consent.

### Outcomes

This study primarily describes cross-sectional findings using population cohort data collected at the 24-month visit, which was chosen because all people living with HIV in the population cohort had a viral load from the blood sample taken during this visit, regardless of whether they were in HIV care or on ART at the time. Analysis of these outcomes was specified in the trial protocol.^17^ In practice, each round of visits took longer than 1 year; therefore, the intended 24-month visit occurred between 30 months and 42 months after trial commencement. Viral suppression data were not collected as part of the CHiP service delivery, but self-reported ART adherence data from the final annual intervention visit, collected at a similar point in time as the 24-month visit data, are presented here. We assessed two main outcomes: first, lack of viral suppression (ie, ≥400 copies of HIV RNA per mL) among people living with HIV who reported currently being on ART (available in population cohort data only); and second, non-adherence to ART, which was defined in population cohort data as reporting having missed one or more pills in the past 7 days among people living with HIV who reported currently being on ART, and defined in CHiP data as reporting having missed one or more pills in the last 3 days among people living with HIV who reported currently being on ART.

In the population cohort and CHiP data, individuals were included if they reported to be HIV-positive and currently being on ART. Additionally, laboratory tests confirming HIV-positive status were required for inclusion in population cohort analyses.

Four research questions were investigated: did the proportion of individuals with each outcome differ in the intervention arms versus the control arm? What was the relationship between self-reported ART adherence and viral suppression among people living with HIV who reported being currently on ART? What individual and household characteristics were associated with each outcome? And, were findings for self-reported ART adherence similar in the population cohort and CHiP data? The first three questions were addressed using population cohort data; the fourth question was addressed using both population cohort and CHiP data.

We also considered a secondary retention-on-ART outcome, among participants who had reported ever taking ART, using a non-standard definition of retention such that that they were currently taking ART, were adherent to treatment, and had not interrupted treatment in the previous year (appendix pp 10–13).

### Statistical analysis

The geometric means of community-level prevalences, from the population cohort data, were presented as arm-specific estimates of prevalence to correspond to our analysis comparing trial arms. To compare outcomes by arm, a two-stage approach was used to estimate the adjusted prevalence ratio.

Stage one was to fit an individual-level logistic regression model with triplet, gender, age group, and an interaction between gender and age group, as explanatory variables, providing an estimate of the expected prevalence in each community under the null hypothesis of no intervention effect.

The second stage was to take the log of the ratio of the observed prevalence and expected prevalence and use it as the outcome for a linear regression with triplet and arm as explanatory variables. The exponential of the two trial arm coefficients (A *vs* C and B *vs* C) provided the estimated prevalence ratio for arm A and arm B compared with arm C. A pooled estimate of intervention effect (combining arm A and arm B) was obtained by taking the mean of the two coefficients and exponentiating it. A sensitivity analysis was performed excluding the participants who had started ART in the past 6 months as the number who have recently initiated ART could differ between trial arms and could also be associated with the outcomes. Subgroup analyses were performed stratifying by age and gender.

A cross-tabulation of self-reported adherence (yes or no) and viral suppression (suppressed *vs* not suppressed) was performed among participants who reported that they were currently on ART using the population cohort data. A logistic regression model—using viral suppression as the outcome and self-reported adherence as an explanatory variable—provided evidence for the strength of this association. Differences between study communities were accounted for by adjusting for community triplet as a fixed effect. The results were also adjusted for age group, gender, recent ART initiation (within the past 6 months), and trial arm.

A logistic regression model was fitted for each outcome, including triplet (to account for differences between communities), age group, gender, and trial arm, then subsequently including all variables (appendix p 2) using the population cohort data. Participants who scored 8 or more on the Alcohol Use Disorders Identification Test (AUDIT) were classified as potentially participating in harmful alcohol consumption.

The prevalence of self-reported adherence in each age and gender subgroup was calculated using CHiP data collected during service delivery in arms A and B. The prevalence (pooled across the two arms) was compared with results from the population cohort, excluding arm C. A p value for this comparison was obtained using a logistic regression with data source (population cohort *vs* CHiP) as the explanatory variable and non-adherence as the outcome, adjusted for gender, age group, and community.

All analyses were performed using Stata 16 and the trial was registered with ClinicalTrials.gov, NCT01900977.

### Role of the funding source

The funder of the study had no role in study design, data collection, data analysis, data interpretation, or writing of the report.

## Results

48 301 individuals aged 18–44 years were enrolled into the population cohort, of whom 34 143 (70·7%) were women. 30 008 (62·1%) of 48 301 individuals had a 24-month visit, with 6259 (20·9%) confirmed as HIV-positive from a blood sample tested at a centralised laboratory. Of those, 4346 (69·4%) of these 6259 reported that they knew their HIV-positive status and disclosed it to the population cohort interviewer; 3626 (83·4%) of those participants reported ever being on ART and 3570 (82·1%) reported being currently on ART (appendix p 3). Among the group of 3570 participants who reported currently being on ART at the 24-month visit, 3171 (88·8%) were women and ages ranged from 18 to 48 years. Overall, 345 (9·7%) of 3566 (four people were missing adherence status) were non-adherent to ART and 416 (11·7%) of 3554 participants were not virally suppressed (16 were missing viral suppression status). Reported non-adherence was similar across men and women and among participants in the two countries. Viral non-suppression was more common among men than women (14·8% *vs* 11·3%) and among participants in South Africa compared with Zambia (16·4% *vs* 9·4%; table 1).

**Table 1:** Participant characteristics

		**All (n=3570)**	**Men (n=399)**	**Women (n=3171)**
**Country**				
Zambia		2365 (66·2%)	290 (72·7%)	2075 (65·4%)
South Africa		1205 (33·8%)	109 (27·3%)	1096 (34·6%)
**Arm**				
A		1139 (31·9%)	126 (31·6%)	1013 (31·9%)
B		1167 (32·7%)	129 (32·3%)	1038 (32·7%)
C		1264 (35·4%)	144 (36·1%)	1120 (35·3%)
**Age group, years**				
18–24		256 (7·2%)	14 (3·5%)	242 (7·6%)
25–29		534 (15·0%)	34 (8·5%)	500 (15·8%)
30–34		864 (24·2%)	82 (20·6%)	782 (24·7%)
35–39		893 (25·0%)	94 (23·6%)	799 (25·2%)
40–48		1023 (28·7%)	175 (43·9%)	848 (26·7%)
**Outcomes**				
Overall				
	Non-adherent* (four MV)	345 (9·7%)	42 (10·5%)	303 (9·6%)
	Not virally suppressed* (16 MV)	416 (11·7%)	59 (14·8%)	357 (11·3%)
Zambia only				
	Non-adherent* (three MV)	226 (9·6%)	31 (10·7%)	195 (9·4%)
	Not virally suppressed* (two MV)	221 (9·4%)	37 (12·8%)	184 (8·9%)
South Africa only				
	Non-adherent* (one MV)	119 (9·9%)	11 (10·1%)	108 (9·9%)
	Not virally suppressed* (14 MV)	195 (16·4%)	22 (20·4%)	173 (16·0%)

Arm A was combination HIV prevention package including immediate antiretroviral therapy (ART) initiation, arm B was combination HIV prevention package excluding immediate ART initiation, and arm C was standard of care. Data are n (%). MV=missing values.

* Percentages among individuals who reported currently being on antiretroviral therapy.

No evidence was found of a difference between arms in viral suppression or self-reported ART adherence after accounting for age, gender, and community triplet (table 2). A sensitivity analysis excluding those who had first started ART in the 6 months before the 24-month visit yielded similar results (appendix p 5). Subanalyses were performed stratifying the analysis by gender and age group, and there was again no evidence of differences between arms within any of these subgroups (appendix p 6).

**Table 2:** Comparison of non-adherence and not being virally suppressed by study arm among individuals who self-reported they were currently on antiretroviral therapy

	Estimated proportion*(95% CI)	**Prevalence ratio vs arm C (95% CI)**	**p value**
**Non-adherent**			
Arm A	9·8% (7·0–13·6)	0·92 (0·64–1·31)	0·60
Arm B	10·0% (7·8–12·8)	0·93 (0·65–1·33)	0·67
Arms A and B (pooled)	9·9% (8·3–11·8)	0·92 (0·68–1·26)	0·59
Arm C	10·9% (7·4–16·0)	1 (ref)	..
**Not virally suppressed**			
Arm A	10·4% (6·2–17·2)	0·80 (0·57–1·12)	0·17
Arm B	12·5% (8·0–19·6)	0·94 (0·67–1·32)	0·72
Arms A and B (pooled)	11·4% (8·5–15·2)	0·87 (0·65–1·16)	0·31
Arm C	13·4% (8·4–21·3)	1 (ref)	..

Arm A was combination HIV prevention package including immediate antiretroviral therapy (ART) initiation, arm B was combination HIV prevention package excluding immediate ART initiation, and arm C was standard of care.

* Proportions provided are the geometric means of community prevalences (arithmetic means are provided in the appendix p 4).

Among the 3570 individuals who reported being currently on ART at the 24-month visit, 3550 had viral load results and reported data on ART adherence. Out of those, 3207 (90·3%) participants reported adherence to ART and 343 (9·7%) reported non-adherence. Among people living with HIV who reported adherence to ART, 364 (11·4%) of 3207 were not virally suppressed compared with 52 (15·2%) of 343 in those reporting non-adherence. The median duration on ART of the participants who were virally suppressed was 3·7 years (IQR 1·9–6·9) and 4·0 years (1·5–7·5) in those not virally suppressed.

After adjustment, participants who were classified as non-adherent to ART had an estimated odds of not being suppressed approximately one-third higher than those classed as adherent, although evidence for an association was weak (odds ratio [OR] 1·35, 95% CI 0·98–1·86; p=0·068). When stratified by gender, there was evidence of an association in men (2·64, 1·23–5·66; p=0·013) but not in women (1·19, 0·83–1·70; p=0·35), although the evidence for effect modification was weak (p=0·070; table 3). This difference in association seen in men and women was observed in both countries, but the difference was greater in Zambia (appendix p 7). The proportion of HIV-positive participants who were not virally suppressed was higher in South Africa than Zambia, in both adherent (15·7% *vs* 9·2%) and non-adherent (23·1% *vs* 11·1%) participant groups (table 3).

**Table 3:** Associations between self-reported adherence to antiretroviral therapy (ART) and viral suppression among individuals who reported currently being on ART (n=3550)

		**Percentage not virally suppressed**		**Unadjusted analysis**		**Adjusted**		
		Adherent	Non-adherent	OR*	p value	OR*†	p value	p_interaction_‡
All		364/3207 (11·4%)	52/343 (15·2%)	1·40 (1·02–1·91)	0·038	1·35 (0·98–1·86)	0·068	..
Gender		..	..	..	..	..	..	0·070
	Men	47/356 (13·2%)	12/42 (28·6%)	2·63 (1·26–5·49)	0·010	2·64 (1·23–5·66)	0·013	..
	Women	317/2851 (11·1%)	40/301 (13·3%)	1·23 (0·86–1·74)	0·26	1·19 (0·83–1·70)	0·35	..
Age, years		..	..	..	..	..	..	0·38
	<30	109/696 (15·7%)	21/89 (23·6%)	1·66 (0·98–2·83)	0·060	1·65 (0·96–2·82)	0·070	..
	≥30	255/2511 (10·2%)	31/254 (12·2%)	1·23 (0·83–1·83)	0·31	1·22 (0·81–1·82)	0·34	..
ART duration		..	..	..	..	..	..	0·74
	<6 months	27/200 (13·5%)	4/25 (16·0%)	1·22 (0·39–3·83)	0·73	1·12 (0·34–3·64)	0·85	..
	≥6 months	337/3007 (11·2%)	48/318 (15·1%)	1·41 (1·02–1·95)	0·040	1·37 (0·98–1·92)	0·064	..
Country		..	..	..	..	..	..	0·45
	Zambia§	196/2134 (9·2%)	25/226 (11·1%)	1·23 (0·79–1·91)	0·36	1·19 (0·76–1·86)	0·44	..
	South Africa§	168/1073 (15·7%)	27/117 (23·1%)	1·62 (1·02–2·56)	0·041	1·53 (0·96–2·44)	0·074	..

Data are n/N (%). OR=odds ratio.

* ORs compare the odds of not being virally suppressed among individuals classified as non-adherent versus adherent.

† Adjusted ORs are adjusted for age group, gender, recent ART initiation (<6 months), trial arm, and community triplet.

‡ p value for interaction tests the hypothesis that the (adjusted) OR is equal in the two subgroups; the smaller the p value, the stronger the evidence that the ORs differ between groups.

§ Adjusted ORs as above, but not adjusted for triplet.

Viral non-suppression was more prevalent among men (59 [14·8%] of 398 *vs* 357 [11·3%] of 3156 in women) and younger age groups. It was highest in the three community triplets in South Africa (triplets 5–7), with the highest prevalence of 24 (24%) of 100 observed in triplet 7, which was also the smallest triplet by population (table 4).

**Table 4:** Associations between participant characteristics and not being virally suppressed among individuals who self-reported they were currently on antiretroviral therapy (ART; N=3554)

		**n**	**Not virally suppressed**	Minimally adjusted*		**Fully adjusted**†	
				OR (95% CI)	p value‡	OR (95% CI)	p value§
Gender (0 MV)		..	..	..	0·0056	..	0·14
	Men	398	59 (14·8%)	1·57 (1·15–2·14)	..	1·35 (0·92–1·98)	..
	Women	3156	357 (11·3%)	1 (ref)	..	1 (ref)	..
Age group, years (0 MV)		..	..	..	<0·0001	..	0·0050
	18–24	256	49 (19·1%)	1 (ref)	..	1 (ref)	..
	25–29	532	81 (15·2%)	0·70 (0·47–1·04)	..	0·66 (0·42–1·04)	..
	30–34	860	109 (12·7%)	0·55 (0·38–0·80)	..	0·53 (0·34–0·83)	..
	35–39	889	84 (9·4%)	0·40 (0·27–0·58)	..	0·43 (0·27–0·68)	..
	40+	1017	93 (9·1%)	0·37 (0·25–0·54)	..	0·44 (0·28–0·70)	..
Community triplet (0 MV)		..	..	..	<0·0001	..	0·0002
	1	454	35 (7·7%)	1 (ref)	..	1 (ref)	..
	2	589	50 (8·5%)	1·07 (0·68–1·69)	..	1·13 (0·65–1·95)	..
	3	532	51 (9·6%)	1·31 (0·83–2·07)	..	1·26 (0·73–2·17)	..
	4	788	85 (10·8%)	1·44 (0·95–2·18)	..	1·49 (0·90–2·46)	..
	5	627	87 (13·9%)	2·08 (1·37–3·15)	..	1·97 (1·16–3·32)	..
	6	464	84 (18·1%)	2·70 (1·77–4·11)	..	3·00 (1·76–5·10)	..
	7	100	24 (24·0%)	3·91 (2·18–7·03)	..	3·23 (1·50–6·96)	..
Wealth quintile (22 MV)		..	..	..	0·28	..	0·20
	1 (lowest)	951	127 (13·4%)	1 (ref)	..	1 (ref)	..
	2	735	85 (11·6%)	0·80 (0·59–1·08)	..	0·77 (0·54–1·10)	..
	3	734	92 (12·5%)	0·91 (0·67–1·23)	..	0·81 (0·57–1·16)	..
	4	714	66 (9·2%)	0·71 (0·51–0·98)	..	0·63 (0·43–0·92)	..
	5 (highest)	398	44 (11·1%)	0·87 (0·59–1·29)	..	0·76 (0·47–1·20)	..
Education level (28 MV)		..	..	..	0·20	..	0·24
	None	74	6 (8·1%)	1·09 (0·46–2·61)	..	1·14 (0·43–3·02)	..
	Grades 1–7	979	82 (8·4%)	1 (ref)	..	1 (ref)	..
	Grades 8–12	2327	313 (13·5%)	1·31 (0·99–1·73)	..	1·38 (0·99–1·92)	..
	College or university	146	13 (8·9%)	0·95 (0·50–1·78)	..	1·06 (0·51–2·22)	..
Marital status (14 MV)		..	..	..	0·49	..	0·82
	Currently	1896	193 (10·2%)	1 (ref)	..	1 (ref)	..
	Never	854	144 (16·9%)	1·11 (0·84–1·47)	..	0·95 (0·68–1·34)	..
	Previously	790	79 (10·0%)	1·17 (0·88–1·55)	..	1·09 (0·76–1·57)	..
Sex partners in the past year (168 MV)		..	..	..	0·68	..	0·37
	None	892	88 (9·9%)	1 (ref)	..	1 (ref)	..
	One	2354	289 (12·3%)	1·16 (0·90–1·51)	..	1·26 (0·90–1·76)	..
	2–4	116	14 (12·1%)	0·99 (0·54–1·83)	..	0·94 (0·45–1·93)	..
	5–9	24	3 (12·5%)	1·24 (0·36–4·31)	..	0·50 (0·06–4·19)	..
Drugs in the past year (7 MV)		..	..	..	0·54	..	0·52
	No	3487	405 (11·6%)	1 (ref)	..	1 (ref)	..
	Yes	60	10 (16·7%)	1·27 (0·61–2·65)	..	0·76 (0·32–1·79)	..
Harmful drinking (0 MV)§		..	..	..	<0·0001	..	<0·0001
	No	3234	343 (10·6%)	1 (ref)	..	1 (ref)	..
	Yes	320	73 (22·8%)	2·25 (1·68–3·02)	..	2·11 (1·50–2·97)	..
Time since ART initiation (473 MV)		..	..	..	0·64	..	0·27
	<3 months	110	19 (17·3%)	1·44 (0·85–2·43)	..	1·55 (0·89–2·71)	..
	3–6 months	116	12 (10·3%)	0·79 (0·42–1·48)	..	0·58 (0·27–1·23)	..
	6–12 months	223	28 (12·6%)	0·99 (0·64–1·52)	..	0·96 (0·61–1·53)	..
	12–24 months	366	46 (12·6%)	1·07 (0·76–1·52)	..	1·12 (0·77–1·61)	..
	>24 months	2266	241 (10·6%)	1 (ref)	..	1 (ref)	..
Any stigma (123 MV)		..	..	..	0·040	..	0·10
	No	2417	273 (11·3%)	1 (ref)	..	1 (ref)	..
	Yes	1014	128 (12·6%)	1·28 (1·01–1·62)	..	1·25 (0·96–1·63)	..
Internal stigma (80 MV)¶		..	..	..	0·056	..	0·049
	No	2961	336 (11·3%)	1 (ref)	..	1 (ref)	..
	Yes	513	68 (13·3%)	1·33 (1·00–1·78)	..	1·40 (1·01–1·95)	..
Community stigma (92 MV)¶		..	..	..	0·036	..	0·34
	No	2740	311 (11·4%)	1 (ref)	..	1 (ref)	..
	Yes	722	93 (12·9%)	1·32 (1·02–1·71)	..	1·17 (0·85–1·60)	..
Health worker stigma (84 MV)¶		..	..	..	0·85	..	0·70
	No	3309	387 (11·7%)	1 (ref)	..	1 (ref)	..
	Yes	161	20 (12·4%)	1·05 (0·64–1·72)	..	0·89 (0·49–1·61)	..

Data are n (%) or OR (95% CI). MV=missing values. OR=odds ratio.

* Adjusted for age category, gender, trial arm, and triplet.

† Adjusted for all other covariates (including any stigma, but not individual stigma elements).

‡ The p value provides the strength of evidence for an association between the exposure and not being virally suppressed, using a likelihood ratio test which tests the null hypothesis that the odds of not being virally suppressed are the same in all categories of the exposure.

‡ Participants who scored 8 or more on the Alcohol Use Disorders Identification Test were classified as potentially participating in harmful alcohol consumption.

¶ Fully adjusted model is adjusted for other stigma elements, but not any stigma.

After adjusting for all other covariates, strong evidence was found that younger individuals (p=0·0050) and those classified as participating in harmful drinking (OR 2·11, 95% CI 1·50–2·97; p<0·0001) had greater odds of not being virally suppressed, with weaker evidence of higher odds among those who had reported internal stigma (1·40, 1·01–1·95; p=0·049). There was also evidence that the odds of non-suppression were higher among men after adjusting only for age, trial arm, and community triplet (OR 1·57, 95% CI 1·15–2·14; p=0·0056), which weakened once fully adjusted (1·35, 0·92–1·98; p=0·14), primarily due to the adjustment for alcohol use, which is likely to lie on the causal pathway to viral suppression. There was little difference between the minimally adjusted and fully adjusted ORs for other variables (table 4).

There was weak evidence that the association between alcohol and viral suppression was different in men and women (p_interaction_=0·069). Among men, there was no evidence of a change in the odds of not being virally suppressed among individuals classified as people who participate in harmful drinking versus those who were not (OR 1·07, 95% CI 0·46–2·51; p=0·86), but among women, there was strong evidence that the odds were higher in people who participate in harmful drinking (2·44, 1·68–3·54; p<0·0001; table 5).

**Table 5:** Stratum-specific associations between not being virally suppressed and gender or alcohol use among individuals who self-reported they were currently on antiretroviral therapy

		**N**	Minimally adjusted*		**Fully adjusted**†	
			OR (95% CI)	p value	OR (95% CI)	p value
Non-harmful drinking‡				0·0023		0·027
	Women	2915	1 (ref)		1 (ref)	
	Men	319	1·70 (1·21–2·40)		1·60 (1·05–2·42)	
Harmful drinking‡				0·29		0·41
	Women	241	1 (ref)		1 (ref)	
	Men	79	0·68 (0·34–1·38)		0·70 (0·30–1·64)	
Women				<0·0001		<0·0001
	Non-harmful drinking‡	2915	1 (ref)		1 (ref)	
	Harmful drinking‡	241	2·70 (1·95–3·72)		2·44 (1·68–3·54)	
Men				0·83		0·86
	Non-harmful drinking‡	319	1 (ref)		1 (ref)	
	Harmful drinking‡	79	1·08 (0·54–2·19)		1·07 (0·46–2·51)	

Data are OR (95% CI). OR=odds ratio.

* Adjusted for age category, gender, trial arm, and community triplet.

† Adjusted for all other covariates.

‡ Participants who scored 8 or more on the Alcohol Use Disorders Identification Test were classified as potentially participating in harmful alcohol consumption.

Repeating the same analysis but with non-adherence as the outcome produced fairly similar results. There was little evidence of association between age or gender and non-adherence, but the associations with stigma indicators were stronger with individuals experiencing stigma (particularly internalised stigma) having greater odds of non-adherence. We also found an association with drug use in the last year, in which the estimated odds for both outcomes were twice as high among individuals who reported having used drugs compared with those who reported no drug use. An interaction between gender and alcohol use was observed similar to that from the analysis of viral suppression (appendix pp 8, 9).

During the final round of the intervention delivery, CHiPs collected data on self-reported adherence from 28 761 residents aged at least 18 years in arm A and B communities who had reported being currently on ART. CHiP data found that 1·1% of individuals aged 18–44 years who reported that they were currently on ART were classified as non-adherent to ART, compared with 9·9% in the population cohort (p<0·0001; appendix p 9)

## Discussion

We found no evidence that introduction of a large-scale universal testing and treatment intervention was associated with lower viral suppression or self-reported ART adherence among individuals on ART; however, in all subgroups analysed, at least 10% of participants currently on ART were not virally suppressed, even when reporting adherence to treatment. This proportion was highest in young people, men, and also individuals reporting harmful alcohol intake, recreational drug use, and stigma.

There was geographical variation across the community triplets for both outcomes and there was a particular difference in viral non-suppression between the two countries, with individuals in South Africa more likely not to be virally suppressed, despite similar levels of self-reported ART adherence. Finally, we obtained much lower estimates of non-adherence in data collected as part of the intervention service delivery compared with the data obtained in the population cohort.

The absence of evidence for a difference in both outcomes between the control and intervention arms can reassure us that universal testing and treatment has not had a large adverse effect on viral suppression and adherence to ART among people living with HIV currently on ART over a period of 3 years. This finding was consistent with results from the ANRS 12249 TasP trial.^18^ The findings here also provide no evidence to suggest that differences in viral suppression or self-reported adherence between the intervention arms (A and B) were responsible for the observed difference in HIV incidence seen in the main trial results.^15^

During the trial, viral suppression among individuals reporting being currently on ART was close to the 90% UNAIDS target (the so-called third 90) but fell short of the 95% goal for 2030. Participants classed as participating in harmful alcohol use were at higher risk of not being virally suppressed (and being non-adherent) and our estimate is consistent with findings from other studies in sub-Saharan Africa.^19^, ^20^, ^21^ This effect seemed more apparent in women than men, which is plausible in this setting given fewer women meet the AUDIT classification of problem drinking of alcohol than men; therefore, heavy drinking among women could be more indicative of other issues than in men.^22^ The sample size for men was small so we should treat this observation with caution; a previous study in Botswana found no difference between men and women in the effect of alcohol on adherence.^23^ We found that viral suppression and adherence were worse among individuals who reported internalised stigma, potentially suggesting that this type of stigma is a barrier to viral suppression even after commencement of treatment, which was consistent with findings previously published that looked at viral suppression among all people living with HIV who knew and disclosed their HIV-positive status.^24^ This association was also observed at baseline before the start of the trial^25^ and therefore appears to be unchanged in the context of universal testing and treatment. In most subgroups, at least 10% were not virally suppressed, suggesting a generalised problem not limited to marginalised groups, illustrating the difficulty reaching the UNAIDS targets. The reason for this non-suppression cannot be certain from our data, but it is likely that drug resistance is a contributory factor.

During the HPTN 071 (PopART) trial, our estimates of non-adherence from intervention service delivery (CHiP) data were very low, and much lower than the estimates obtained from the population cohort. A small amount of that could be due to the differences in the definition of adherence (missed pills in last 3 days *vs* 7 days), but that is unlikely to be a large contributory factor. The intervention data are only from the individuals who agreed to participate in the intervention, who could have been more likely to adhere to treatment than those who did not participate, which, coupled with the gaps between self-reported adherence and viral suppression observed in the population cohort data, reinforces the need to perform regular viral load monitoring.

A strength of this study was that it had a very large sample of people living with HIV, randomly selected from a range of communities across two countries, which helps with generalisability and reliability of findings. However, there were limitations in this study. The first was the small number of men in the sample, limiting our ability to make inferences about men. People living with HIV on ART who did not disclose their HIV or ART status to investigators could not be included in our analysis, and individuals who did not disclose may be different in terms of adherence or viral suppression to those who were willing to disclose their status. The data on ART adherence were self-reported so could have been prone to recall and social desirability bias. For the risk factor analyses, both the exposures and outcome were measured at the same point in time, so any causal interpretations of the results should be made with care. Finally, it should be noted that not finding evidence of a difference between trial arms does not prove that there is no difference as the study was powered to show differences rather than non-inferiority. However, the point estimates were in the direction of arms A and B having better outcomes than the control arm C, and the upper end of the CIs are 1·16 (for non-viral suppression) and 1·26 (for non-adherence), which gives us confidence that there was no substantial adverse effect due to the intervention.

In conclusion, there was no evidence to suggest that people living with HIV on ART from communities that had received 3 years of a universal testing and treatment intervention are less likely to be virally suppressed or report non-adherence compared with those in communities that had not received the intervention.

## Data sharing

Data collected for the study will be made available to others. A complete de-identified dataset of the population cohort and community HIV care providers datasets will be available on request at the aggregate level. Individual line data cannot be shared due to ethics, consent, and data ownership issues.

## Declaration of interests

AM, EP-M, and SHE report funding from the National Institutes of Health (NIH). DD and TS reports funding from the National Institute of Allergy and Infectious Diseases (NIAID) and NIH. DM, SFl, and HA report funding from NIH, the US President's Emergency Plan for AIDS Relief (PEPFAR), the Bill & Melinda Gates Foundation, and the International Initiative for Impact Evaluation. SFid reports participation on a Data and Safety Monitoring Board for the Disasters Emergency Committee HIV vaccine trial. GH reports funding from NIH, NIAID, and the National Institute of Mental Health, the Gates Foundation, South African TB Think Tank, the United States Agency for International Development, Unitaid, Stop TB partnership, and participation on an advisory board for Templeton World Charity Foundation. All other authors declare no competing interests.
