# Supplementary material for: Viral suppression and self-reported ART adherence after 3 years of universal testing and treatment in the HPTN 071 (PopART) community-randomised trial in Zambia and South Africa: a cross-sectional analysis
Source: Lancet HIV. 2022 Nov 1;9(11):e751–9. doi: 10.1016/S2352-3018(22)00237-5 (PMC9646982; doi:10.1016/S2352-3018(22)00237-5)
Supplement: Supplementary appendix [file mmc1.pdf]

THE LANCET
HIV

Supplementary appendix

This appendix formed part of the original submission and has been peer reviewed. We post it as supplied by the authors.

Supplement to: Macleod D, Shanaube K, Skalland T, et al. Viral suppression and self-reported ART adherence after 3 years of universal testing and treatment in the HPTN 071 (PopART) community-randomised trial in Zambia and South Africa: a cross-sectional analysis. *Lancet HIV* 2022; **9:** e751–59.

Viral suppression and self-reported ART adherence after three years of universal testing and treatment in the HPTN 071 (PopART) community-randomized trial in Zambia and South Africa

Appendix

Table of Contents

Supplementary material S1: Explanatory variables assessed during risk factor analysis 2

Supplementary Figure S2: Inclusion criteria for this study (PC data) 3

Supplementary Table S3: Arithmetic means of cluster prevalences of non-adherence and non-viral suppression among those currently on ART 4

Supplementary Table S4: Comparison of non-adherence and not being virally suppressed, restricted to only those who reported that they were on ART for longer than six months, by study arm 5

Supplementary Table S5: Comparison of non-adherence and not being virally suppressed, by study arm and stratified by gender 6

Supplementary Table S6: Comparison of non-adherence and not being virally suppressed, by study arm and stratified by age group 6

Supplementary Table S7: Associations between self-reported ART adherence and viral suppression among those who reported currently being on ART. Data are stratified by country and also stratified by gender, age group and whether reported first starting ART in the past six months. 7

Supplementary Table S8: Associations between participant characteristics and non-adherence (N=3,566) 8

Supplementary Table S9: Stratum-specific associations between non-adherence and gender or alcohol use 9

Supplementary Table S10: Proportion of individuals (age 18+) who were classified as non-adherent to ART, based on data collected by CHiPs during intervention delivery; data are compared to equivalent data from the PC. 9

Supplementary material S11: Analyses of retention 10

Supplementary material S12: Members of the HPTN 071 (PopART) trial study team 14

Supplementary material S1: Explanatory variables assessed during risk factor analysis

Variables assessed included: study arm, community, triplet, age group, gender, wealth quintile, education level, marital status, number of sex partners in the past 12 months, time since first ART initiation, recreational drug use in the past 12 months, potentially harmful alcohol consumption (defined as a score of 8+ on the Alcohol Use Disorders Identification Test – AUDIT), and experiences of HIV-related stigma.

Wealth quintile was obtained by creating a wealth score using a principal component analysis based on assets and housing quality within each country separately. Then within each country the score is categorised into five groups of equal size based on the wealth score.

Three domains of stigma were measured; current internalised stigma, having ever experienced community stigma and having ever experienced health setting stigma. Three questions were asked for each domain, with each measured on 5-point Likert scales. If participants reported experiencing stigma on one or more of these questions they were classed as having experienced that type of stigma. A composite measure of stigma was also defined for participants who experienced stigma in any of the three domains. Further detail on these stigma domains can be found in the paper by Hargreaves et al.[28]

Supplementary Figure S2: Inclusion criteria for this study (PC data)

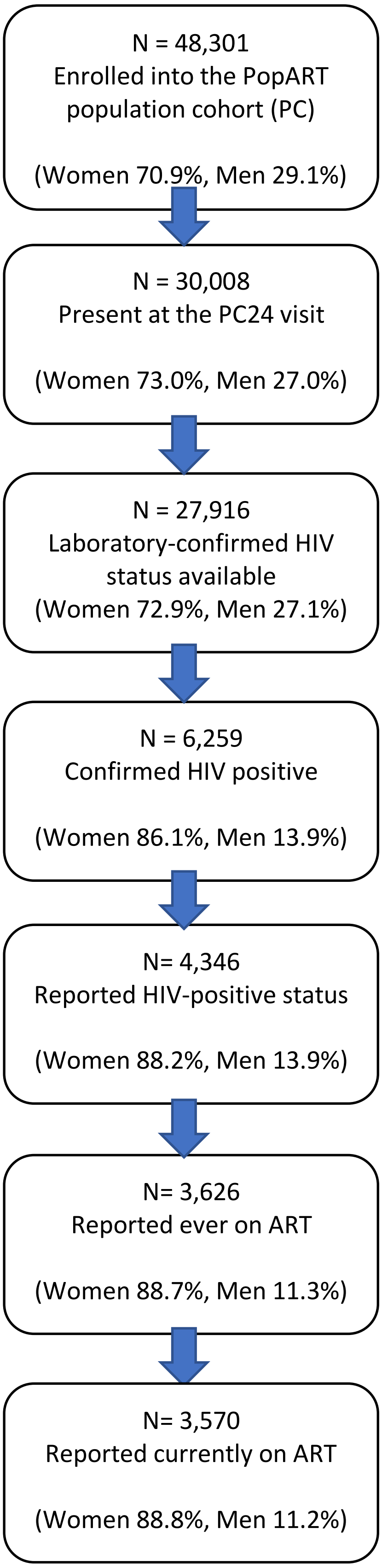

Abbreviations:
ART: antiretroviral therapy; PC: Population Cohort.

Supplementary Table S3: Arithmetic means of cluster prevalences of non-adherence and non-viral suppression among those currently on ART

Trial arm	Arithmetic mean proportion* (95% CI)
Not adherent (among those currently on ART)	
A	10.4% (6.3%-14.5%)
B	10.3% (7.8%-12.8%)
A & B (pooled)	10.3% (8.3%-12.4%)
C	11.7% (7.2%-16.2%)
Not virally suppressed (among those currently on ART)	
A	11.6% (6.7%-16.4%)
B	13.9% (7.0%-20.7%)
A & B (pooled)	12.7% (9.1%-16.3%)
C	15.0% (7.5%-22.4%)

Abbreviations: ART: antiretroviral therapy

*Estimate is the arithmetic mean of the cluster prevalences, 95% CI based on variation between communities

Supplementary Table S4: Comparison of non-adherence and not being virally suppressed, restricted to only those who reported that they were on ART for longer than six months, by study arm

Trial arm	Estimated proportion* (95% CI)	Prevalence ratio vs arm C (95% CI)	p-value
Non-adherent (among those currently on ART)			
A	9.6% (6.9%-13.2%)	0.89 (0.61-1.31)	0.52
B	9.4% (7.0%-12.6%)	0.87 (0.59-1.28)	0.45
A & B (pooled)	9.5% (7.9%-11.4%)	0.88 (0.63-1.23)	0.42
C	11.0% (7.4%-16.2%)		
Not virally suppressed (among those currently on ART)			
A	10.4% (6.1%-18.0%)	0.80 (0.56-1.15)	0.21
B	11.8% (7.8%-18.1%)	0.90 (0.63-1.28)	0.52
A & B (pooled)	11.1% (8.3%-14.9%)	0.85 (0.62-1.16)	0.27
C	13.5% (8.5%-21.3%)		

Abbreviations: ART: antiretroviral therapy

Supplementary Table S5: Comparison of non-adherence and not being virally suppressed, by study arm and stratified by gender

	**Men**			**Women**		
Trial arm	Estimated proportion* (95% CI)	Prevalence ratio vs arm C (95% CI)	p-value	Estimated proportion* (95% CI)	Prevalence ratio vs arm C (95% CI)	p-value
Non-adherent (Among those currently on ART)						
A	15.7% (6.6%-37.8%)	0.97 (0.40-2.34)	0.95	9.2% (6.1%-13.7%)	0.94 (0.53-1.64)	0.80
B	11.5% (6.2%-21.4%)	0.75 (0.31-1.80)	0.49	8.7% (5.7%-13.4%)	0.89 (0.51-1.56)	0.66
A & B (pooled)	13.3% (8.7%-20.3%)	0.85 (0.40-1.82)	0.66	8.9% (6.9%-11.5%)	0.91 (0.56-1.48)	0.69
C	15.4% (4.9%-48.2%)			10.0% (7.4%-13.5%)		
Not virally suppressed (Among those currently on ART)						
A	14.9% (6.3%-35.2%)	0.67 (0.37-1.20)	0.16	8.6% (3.6%-20.4%)	0.72 (0.39-1.32)	0.26
B	13.4% (7.1%-25.3%)	0.51 (0.28-0.92)	0.028	12.2% (7.9%-18.8%)	1.03 (0.56-1.89)	0.91
A & B (pooled)	14.0% (9.2%-21.3%)	0.58 (0.35-0.97)	0.039	10.2% (6.7%-15.6%)	0.86 (0.51-1.46)	0.55
C	27.0% (12.7%-57.5%)			12.2% (8.1%-18.5%)		

Supplementary Table S6: Comparison of non-adherence and not being virally suppressed, by study arm and stratified by age group

	**Less than 30 years old**			**30+ years old**		
Trial arm	Estimated proportion* (95% CI)	Prevalence ratio vs arm C (95% CI)	p-value	Estimated proportion* (95% CI)	Prevalence ratio vs arm C (95% CI)	p-value
Non-adherent (Among those currently on ART)						
A	11.1% (6.3%-19.5%)	0.90 (0.50-1.61)	0.69	9.3% (6.7%-12.8%)	0.81 (0.48-1.36)	0.39
B	11.9% (8.1%-17.4%)	0.96 (0.53-1.72)	0.87	9.1% (6.2%-13.3%)	0.79 (0.47-1.32)	0.34
A & B (pooled)	11.5% (8.6%-15.3%)	0.93 (0.56-1.54)	0.75	9.2% (7.4%-11.3%)	0.80 (0.51-1.25)	0.29
C	10.8% (5.8%-20.1%)			11.5% (6.4%-20.9%)		
Not virally suppressed (Among those currently on ART)						
A	15.8% (7.7%-32.4%)	1.13 (0.69-1.84)	0.61	9.0% (5.7%-14.2%)	0.70 (0.50-0.97)	0.034
B	19.7% (11.5%-33.9%)	1.35 (0.83-2.20)	0.21	10.5% (6.7%-16.5%)	0.81 (0.59-1.13)	0.20
A & B (pooled)	17.7% (12.0%-26.0%)	1.23 (0.81-1.89)	0.31	9.7% (7.4%-12.8%)	0.75 (0.57-1.00)	0.050
C	14.5% (8.7%-24.2%)			12.9% (7.4%-22.5%)		

Abbreviations: ART: antiretroviral therapy

Supplementary Table S7: Associations between self-reported ART adherence and viral suppression among those who reported currently being on ART. Data are stratified by country and also stratified by gender, age group and whether reported first starting ART in the past six months.

Zambia

Category	Percentage not virally suppressed		Unadjusted OR	p-value	Adjusted OR*†	p-value	p-value for interaction
	Adherent	Non-adherent					
All	196/2134 (9.2%)	25/201 (11.1%)	1.23 (0.79-1.91)	0.36	1.19 (0.76-1.86)	0.44	N/A
Men	29/259 (11.2%)	8/31 (25.8%)	2.76 (1.13-6.73)	0.026	2.95 (1.19-7.30)	0.019	0.045
Women	167/1875 (8.9%)	17/195 (8.7%)	0.98 (0.58-1.65)	0.93	0.98 (0.58-1.66)	0.94	
Age <30 years	57/489 (11.7%)	10/59 (16.9%)	1.55 (0.74-3.22)	0.24	1.56 (0.74-3.27)	0.24	0.46
Age 30+ years	139/1645 (8.4%)	15/167 (9.0%)	1.07 (0.61-1.87)	0.81	1.10 (0.62-1.92)	0.75	
ART <6 months	15/126 (11.9%)	1/17 (5.9%)	0.46 (0.06-3.74)	0.47	0.51 (0.06-4.17)	0.53	0.34
ART 6+ months	181/2008 (9.0%)	24/209 (11.5%)	1.31 (0.83-2.06)	0.24	1.31 (0.83-2.08)	0.24	

South Africa

Category	Percentage not virally suppressed		Unadjusted OR	p-value	Adjusted OR*†	p-value	p-value for interaction
	Adherent	Non-adherent					
All	168/1073 (15.7%)	27/117 (23.1%)	1.62 (1.02-2.56)	0.041	1.53 (0.96-2.44)	0.074	N/A
Men	18/97 (18.6%)	4/11 (36.4%)	2.52 (0.66-9.49)	0.18	2.10 (0.53-8.26)	0.29	0.61
Women	150/976 (15.4%)	23/106 (21.7%)	1.53 (0.93-2.50)	0.093	1.43 (0.86-2.37)	0.17	
Age <30 years	52/207 (25.1%)	11/30 (36.7%)	1.73 (0.77-3.86)	0.18	1.72 (0.76-3.89)	0.19	0.68
Age 30+ years	116/866 (13.4%)	16/87 (18.4%)	1.46 (0.82-2.59)	0.20	1.39 (0.77-2.50)	0.27	
ART <6 months	12/74 (16.2%)	3/8 (37.5%)	3.10 (0.65-14.7)	0.15	2.39 (0.47-12.17)	0.29	0.56
ART 6+ months	156/999 (15.6%)	24/109 (22.0%)	1.53 (0.94-2.48)	0.087	1.43 (0.87-2.35)	0.16	

*Odds ratios compare the odds of not being virally suppressed among those classified as non-adherent vs. adherent.

†Adjusted odds ratios are adjusted for age group, gender, recent ART initiation (<6 months), trial arm and community triplet.

Abbreviations: OR: odds ratio: N/A: not applicable; ART: antiretroviral therapy.

Supplementary Table S8: Associations between participant characteristics and non-adherence (N=3,566)

Exposure	Category	N	Not adherent n (%)	Minimally Adjusted* OR (95% CI)	Minimally Adjusted* p-value	Fully adjusted† OR (95% CI)	Fully adjusted† p-value
**Sex (0 MV)**	Men	399	42 (10.5%)	1.16 (0.82-1.64)	0.41	0.77 (0.49-1.22)	0.26
	Women	3167	303 (9.6%)	Reference		Reference	
**Age group (0 MV)**	18-24	255	33 (12.9%)	Reference	0.26	Reference	0.55
	25-29	532	56 (10.5%)	0.78 (0.49-1.24)		0.80 (0.48-1.34)	
	30-34	864	81 (9.4%)	0.68 (0.44-1.05)		0.66 (0.40-1.08)	
	35-39	893	86 (9.6%)	0.69 (0.45-1.07)		0.75 (0.45-1.24)	
	40+	1022	89 (8.7%)	0.62 (0.40-0.95)		0.69 (0.41-1.15)	
**Community triplet (0 MV)**	1	456	59 (12.9%)	Reference	0.013	Reference	0.0026
	2	589	55 (9.3%)	0.69 (0.47-1.02)		0.61 (0.38-0.99)	
	3	530	54 (10.2%)	0.78 (0.52-1.15)		0.74 (0.46-1.17)	
	4	787	58 (7.4%)	0.54 (0.37-0.79)		0.38 (0.23-0.61)	
	5	635	64 (10.1%)	0.78 (0.53-1.14)		0.55 (0.33-0.90)	
	6	469	39 (8.3%)	0.61 (0.40-0.94)		0.47 (0.27-0.81)	
	7	100	16 (16.0%)	1.34 (0.73-2.46)		0.90 (0.40-2.04)	
**Wealth quintile (22 MV)**	1 (Lowest)	953	101 (10.6%)	Reference	0.049	Reference	0.17
	2	736	73 (9.9%)	0.87 (0.63-1.21)		0.87 (0.59-1.28)	
	3	739	74 (10.0%)	0.86 (0.62-1.19)		0.78 (0.53-1.15)	
	4	716	54 (7.5%)	0.57 (0.40-0.83)		0.59 (0.38-0.90)	
	5 (Highest)	400	42 (10.5%)	0.75 (0.49-1.13)		0.73 (0.45-1.19)	
**Education level (29 MV)**	None	74	9 (12.2%)	1.58 (0.76-3.32)	0.50	1.27 (0.51-3.13)	0.29
	Grades 1-7	979	82 (8.4%)	Reference		Reference	
	Grades 8-12	2336	239 (10.2%)	1.18 (0.89-1.57)		1.39 (0.99-1.95)	
	College/Uni	148	14 (9.5%)	1.03 (0.55-1.90)		1.24 (0.60-2.54)	
**Marital status (14 MV)**	Currently	1902	178 (9.4%)	Reference	0.81	Reference	0.79
	Never	858	93 (10.8%)	1.11 (0.81-1.53)		1.05 (0.71-1.55)	
	Previously	792	73 (9.2%)	1.00 (0.75-1.34)		0.90 (0.62-1.31)	
**Sex partners in last year (170 MV)**	None	896	70 (7.8%)	Reference	0.0022	Reference	0.20
	One	2360	236 (10.0%)	1.30 (0.98-1.72)		1.23 (0.86-1.77)	
	2-4	116	17 (14.7%)	2.01 (1.12-3.60)		1.30 (0.62-2.71)	
	5-9	24	7 (29.2%)	5.59 (2.19-14.26)		4.27 (1.12-16.28)	
**Drugs in last year (7 MV)**	No	3498	330 (9.4%)	Reference	0.0025	Reference	0.061
	Yes	61	14 (23.0%)	2.93 (1.54-5.56)		2.08 (1.00-4.30)	
**Harmful drinking (0 MV)**	No	3243	284 (8.8%)	Reference	<0.0001	Reference	<0.0001
	Yes	323	61 (18.9%)	2.37 (1.73-3.24)		2.19 (1.52-3.15)	
**Time since ART initiation (471 MV)**	<3 months	110	13 (11.8%)	1.23 (0.67-2.25)	0.49	1.15 (0.58-2.28)	0.48
	3-6 months	115	12 (10.4%)	1.01 (0.54-1.90)		1.14 (0.58-2.21)	
	6-12 months	225	27 (12.0%)	1.25 (0.81-1.93)		1.34 (0.84-2.13)	
	12-24 months	369	30 (8.1%)	0.78 (0.52-1.17)		0.79 (0.51-1.20)	
	24+ months	2276	213 (9.4%)	Reference		Reference	
**Any stigma (123 MV)**	No	2425	203 (8.4%)	Reference	0.0002	Reference	0.0023
	Yes	1018	124 (12.2%)	1.61 (1.26-2.05)		1.53 (1.17-2.01)	
Internal stigma (80 MV) ‡	No	2973	262 (8.8%)	Reference	0.0002	Reference	0.065
	Yes	513	71 (13.8%)	1.76 (1.32-2.35)		1.38 (0.99-1.94)	
Community stigma (92 MV) ‡	No	2748	242 (8.8%)	Reference	0.0036	Reference	0.055
	Yes	726	88 (12.1%)	1.49 (1.15-1.94)		1.37 (1.00-1.89)	
Health worker stigma (84 MV) ‡	No	3320	310 (9.3%)	Reference	0.15	Reference	0.43
	Yes	162	21 (13.0%)	1.44 (0.89-2.32)		1.25 (0.73-2.17)	

* Adjusted for age category, sex, trial arm and triplet † Adjusted for all other covariates (including any stigma, but not individual stigma elements) ‡ Adjusted for other stigma elements, but not any stigma. Abbreviations: MV: Missing values; OR: odds ratio.

Supplementary Table S9: Stratum-specific associations between non-adherence and gender or alcohol use

Strata	Category	Minimally Adjusted*		Fully adjusted†	
		OR (95% CI)	p-value	OR (95% CI)	p-value
Non-harmful drinking	Women	Reference	0.42	Reference	0.87
	Men	1.18 (0.79-1.75)		0.96 (0.59-1.57)	
Harmful drinking	Women	Reference	0.19	Reference	0.054
	Men	0.62 (0.30-1.27)		0.42 (0.18-1.02)	
Women	Non-harmful drinking	Reference	<0.0001	Reference	<0.0001
	Harmful drinking	2.68 (1.90-3.77)		2.50 (1.69-3.69)	
Men	Non-harmful drinking	Reference	0.37	Reference	0.84
	Harmful drinking	1.41 (0.67-2.96)		1.10 (0.44-2.73)	

* Adjusted for age, sex, trial arm and triplet
† Adjusted for all other covariates
Abbreviations: OR: odds ratio.

Supplementary Table S10: Proportion of individuals (age 18+) who were classified as non-adherent to ART, based on data collected by CHiPs during intervention delivery; data are compared to equivalent data from the PC.

		CHiP data<br>Non-adherent to ART<br>(among those currently on ART)		PC data*<br>Non-adherent to ART<br>(among those currently on ART)	
		Men	Women	Men	Women
Age group	18-24	6/248 (2.4%)	24/1,820 (1.3%)	2/9 (22.2%)	19/151 (12.6%)
	25-29	6/391 (1.5%)	45/3,226 (1.4%)	2/21 (9.5%)	35/336 (10.4%)
	30-34	23/1,001 (2.3%)	37/4,568 (0.8%)	7/57 (12.3%)	44/547 (8.0%)
	35-39	12/1,430 (0.8%)	31/4,310 (0.7%)	6/58 (10.3%)	47/533 (8.8%)
	40-44	19/1,512 (1.3%)	28/3,308 (0.8%)	9/87 (10.3%)	38/460 (8.3%)
	45-54	28/1,849 (1.5%)	26/3,223 (0.8%)		
	55-64	6/591 (1.0%)	6/934 (0.6%)		
	65+	0/149 (0.0%)	1/201 (0.5%)		

*Restricted to arms A and B only as CHiP data only available for arms A and B. PC only recruited participants aged 18-44.

Abbreviations: ART: antiretroviral therapy; PC: Population Cohort.

Supplementary material S11: Analyses of retention

As data were collected on whether an individual had ever started ART, we were also able to provide data on a “retention” outcome, defined as the proportion who were currently adherent to ART (and also had not interrupted treatment in last year) among all those who reported ***ever*** starting ART. This outcome was defined as follows:

- Non-retention on ART
  - Defined in **PC data** as reporting not currently being on ART, or being on ART but missing one or more pills in the last *seven* days, or having stopped ART for >1 month at any point in the last 12 months, among PLHIV who reported ***ever*** being on ART
  - Defined in **CHiP data** as reporting not currently being on ART, or being on ART but missing one or more pills in the last *three* days, among PLHIV who reported ***ever*** being on ART. (There was no question asked about >1-month stoppages)

The same analyses for viral suppression and adherence were repeated using this outcome, and the results are shown below:

Supplementary table S11a: Proportions not-retained overall, and by gender and country among all PC participants who reported ever being on ART

		All (N=3,626)	Men (N=411)	Women (N=3,215)
Overall	Not retained (10 MV)	473 (13.0%)	64 (15.6%)	409 (12.7%)
Zambia only	Not retained (7 MV)	290 (12.2%)	40 (13.7%)	250 (12.0%)
SA only	Not retained (3 MV)	183 (14.7%)	24 (20.3%)	159 (14.1%)

Abbreviations: MV: Missing values; SA: South Africa

Supplementary table S11b: Comparison of non-retention by study arm

Trial arm	Estimated proportion* (95% CI)	Prevalence ratio vs. arm C (95% CI)	p-value
Not retained (among those ever on ART)			
A	13.7% (9.7%-19.3%)	0.92 (0.64-1.32)	0.62
B	12.9% (9.3%-17.9%)	0.87 (0.60-1.24)	0.40
A & B (pooled)	13.3% (10.9%-16.3%)	0.89 (0.65-1.22)	0.44
C	15.1% (10.3%-22.0%)		

*Proportions provided are the geometric means of community prevalences (arithmetic means are provided in Supplementary Table S9d).

Abbreviations: CI: confidence intervals

Supplementary table S11c: Comparison of-retention, restricted to only those who reported that they were on ART for longer than six months, by study arm

Trial arm	Estimated proportion* (95% CI)	Prevalence ratio vs arm C (95% CI)	p-value
Not retained (among those ever on ART)			
A	13.5% (9.5%-19.3%)	0.89 (0.61-1.31)	0.54
B	12.5% (8.7%-17.8%)	0.82 (0.56-1.20)	0.28
A & B (pooled)	13.0% (10.5%-16.1%)	0.86 (0.61-1.19)	0.33
C	15.5% (10.6%-22.6%)		

Supplementary table S11d: Arithmetic means of cluster prevalences for non-retention

Trial arm	Arithmetic mean proportion* (95% CI)
Not retained (among those ever on ART)	
A	14.6% (8.6%-20.6%)
B	13.6% (9.4%-17.8%)
A & B (pooled)	14.1% (11.0%-17.2%)
C	16.3% (9.6%-22.9%)

Supplementary table S11e: Comparison of non-retention, by study arm and stratified by gender

	**Men**			**Women**		
Trial arm	Estimated proportion* (95% CI)	Prevalence ratio vs arm C (95% CI)	p-value	Estimated proportion* (95% CI)	Prevalence ratio vs arm C (95% CI)	p-value
Not retained (among those ever on ART)						
A	16.7% (7.1%-39.5%)	0.71 (0.37-1.36)	0.27	13.0% (9.0%-18.8%)	0.95 (0.58-1.55)	0.83
B	13.1% (6.5%-26.3%)	0.58 (0.30-1.10)	0.087	11.6% (8.1%-16.7%)	0.85 (0.52-1.38)	0.47
A & B (pooled)	14.6% (9.2%-23.2%)	0.64 (0.37-1.12)	0.11	12.3% (9.9%-15.3%)	0.90 (0.59-1.37)	0.59
C	22.5% (11.7%-43.0%)			13.9% (10.0%-19.4%)		

Supplementary table S11f: Comparison of non-retention, by study arm and stratified by age group

	**Less than 30 years old**			**30+ years old**		
Trial arm	Estimated proportion* (95% CI)	Prevalence ratio vs arm C (95% CI)	p-value	Estimated proportion* (95% CI)	Prevalence ratio vs arm C (95% CI)	p-value
Not retained (among those ever on ART)						
A	15.9% (9.4%-26.9%)	1.03 (0.61-1.76)	0.89	12.9% (9.2%-18.2%)	0.86 (0.53-1.39)	0.50
B	16.1% (10.1%-25.8%)	1.04 (0.61-1.77)	0.88	11.5% (7.4%-18.1%)	0.77 (0.47-1.24)	0.25
A & B (pooled)	16.0% (11.9%-21.6%)	1.04 (0.65-1.64)	0.87	12.2% (9.6%-15.6%)	0.81 (0.53-1.23)	0.29
C	15.6% (10.0%-24.2%)			15.0% (9.2%-24.4%)		

Abbreviations: ART: antiretroviral therapy

Supplementary table S11g: Associations between participant characteristics and non-retention (N=3,566)

Exposure	Category	N	Not retained n (%)	Minimally Adjusted*		Fully adjusted†	
				OR (95% CI)	p-value	OR (95% CI)	p-value
**Sex (0 MV)**	Men	411	64 (15.6%)	1.32 (0.99-1.78)	0.065	0.98 (0.68-1.42)	0.92
	Women	3205	409 (12.8%)	Reference		Reference	
**Age group (0 MV)**	18-24	260	44 (16.9%)	Reference	0.097	Reference	0.33
	25-29	542	80 (14.8%)	0.82 (0.55-1.23)		0.75 (0.48-1.19)	
	30-34	872	112 (12.8%)	0.68 (0.47-1.00)		0.65 (0.42-1.00)	
	35-39	902	111 (12.3%)	0.65 (0.44-0.95)		0.65 (0.41-1.02)	
	40+	1040	126 (12.1%)	0.62 (0.43-0.91)		0.64 (0.41-1.00)	
**Community triplet (0 MV)**	1	460	74 (16.1%)	Reference	0.0003	Reference	0.0001
	2	589	65 (11.0%)	0.65 (0.45-0.93)		0.52 (0.34-0.82)	
	3	537	74 (13.8%)	0.85 (0.60-1.21)		0.79 (0.52-1.20)	
	4	786	77 (9.8%)	0.56 (0.40-0.79)		0.41 (0.27-0.63)	
	5	648	91 (14.0%)	0.89 (0.64-1.24)		0.71 (0.46-1.09)	
	6	490	67 (13.7%)	0.83 (0.58-1.18)		0.66 (0.41-1.05)	
	7	106	25 (23.6%)	1.66 (0.98-2.79)		1.50 (0.78-2.90)	
**Wealth quintile (22 MV)**	1 (Lowest)	975	143 (14.7%)	Reference	0.010	Reference	0.025
	2	749	104 (13.9%)	0.86 (0.65-1.14)		0.81 (0.59-1.13)	
	3	743	94 (12.7%)	0.75 (0.56-1.01)		0.67 (0.47-0.94)	
	4	724	75 (10.4%)	0.57 (0.41-0.78)		0.56 (0.39-0.80)	
	5 (Highest)	403	55 (13.6%)	0.71 (0.49-1.03)		0.67 (0.44-1.03)	
**Education level (29 MV)**	None	75	12 (16.0%)	1.59 (0.83-3.06)	0.54	1.49 (0.70-3.21)	0.33
	Grades 1-7	992	112 (11.3%)	Reference		Reference	
	Grades 8-12	2370	328 (13.8%)	1.11 (0.87-1.43)		1.28 (0.95-1.73)	
	College/Uni	150	20 (13.3%)	1.04 (0.62-1.77)		1.09 (0.57-2.07)	
**Marital status (15 MV)**	Currently	1919	239 (12.5%)	Reference	0.92	Reference	0.97
	Never	883	135 (15.3%)	1.05 (0.80-1.39)		1.01 (0.72-1.41)	
	Previously	799	97 (12.1%)	1.02 (0.79-1.32)		1.04 (0.75-1.44)	
**Sex partners in last year (173 MV)**	None	910	95 (10.4%)	Reference	0.0006	Reference	0.16
	One	2388	324 (13.6%)	1.30 (1.01-1.66)		1.30 (0.95-1.78)	
	2-4	121	25 (20.7%)	2.12 (1.29-3.51)		1.64 (0.89-3.01)	
	5-9	24	8 (33.3%)	4.91 (2.00-12.05)		2.90 (0.79-10.69)	
**Drugs in last year (7 MV)**	No	3542	452 (12.8%)	Reference	0.0005	Reference	0.019
	Yes	67	20 (29.9%)	2.88 (1.64-5.06)		2.18 (1.17-4.07)	
**Harmful drinking (0 MV)**	No	3280	388 (11.8%)	Reference	<0.0001	Reference	0.0002
	Yes	336	85 (25.3%)	2.39 (1.82-3.15)		1.91 (1.38-2.66)	
**Time since ART initiation (482 MV)**	<3 months	109	13 (11.9%)	0.85 (0.47-1.56)	0.54	0.79 (0.40-1.55)	0.43
	3-6 months	115	13 (11.3%)	0.78 (0.43-1.43)		0.78 (0.40-1.50)	
	6-12 months	227	36 (15.9%)	1.19 (0.81-1.75)		1.28 (0.85-1.93)	
	12-24 months	374	44 (11.8%)	0.83 (0.58-1.17)		0.83 (0.58-1.20)	
	24+ months	2309	296 (12.8%)	Reference		Reference	
**Any stigma (124 MV)**	No	2449	275 (11.2%)	Reference	<0.0001	Reference	0.0007
	Yes	1043	175 (16.8%)	1.72 (1.39-2.12)		1.52 (1.20-1.93)	
Internal stigma (81 MV) ‡	No	3006	356 (11.8%)	Reference	<0.0001	Reference	0.011
	Yes	529	100 (18.9%)	1.87 (1.45-2.40)		1.48 (1.10-1.99)	
Community stigma (91 MV) ‡	No	2780	332 (11.9%)	Reference	0.0001	Reference	0.061
	Yes	745	125 (16.8%)	1.59 (1.27-2.00)		1.31 (0.99-1.74)	
Health worker stigma (83 MV) ‡	No	3364	427 (12.7%)	Reference	0.058	Reference	0.40
	Yes	169	31 (18.3%)	1.51 (1.00-2.26)		1.24 (0.76-2.00)	

* Adjusted for age category, sex, trial arm and triplet † Adjusted for all other covariates (including any stigma, but not individual stigma elements) ‡ Fully adjusted model is adjusted for other stigma elements, but not any stigma. Abbreviations: MV: Missing values; OR: odds ratio.

Supplementary table S11h: Stratum-specific associations between non-retention and gender or alcohol use

Strata	Category	Minimally Adjusted*		Fully adjusted†	
		OR (95% CI)	p-value	OR (95% CI)	p-value
**Non-harmful drinking**	**Women**	Reference	0.091	Reference	0.37
	**Men**	1.34 (0.96-1.87)		1.20 (0.80-1.79)	
**Harmful drinking**	**Women**	Reference	0.29	Reference	0.075
	**Men**	0.72 (0.39-1.33)		0.50 (0.23-1.07)	
**Women**	**Non-harmful drinking**	Reference	<0.0001	Reference	<0.0001
	**Harmful drinking**	2.72 (2.00-3.68)		2.25 (1.58-3.21)	
**Men**	**Non-harmful drinking**	Reference	0.23	Reference	0.86
	**Harmful drinking**	1.47 (0.79-2.73)		0.93 (0.43-2.03)	

* Adjusted for age, sex, trial arm and triplet
† Adjusted for all other covariates
Abbreviations: OR: odds ratio

Supplementary table S11i: Proportion of individuals (age 18+) who were classified as not-retained on ART, based on data collected by CHiPs during intervention delivery; data are compared to data from the PC.

		CHiP data Not retained on ART (among those ever on ART)		PC data* Not retained on ART (among those ever on ART)	
		Men	Women	Men	Women
**Age group**	**18-24**	19/261 (7.3%)	146/1,942 (7.5%)	2/9 (22.2%)	28/155 (18.1%)
	**25-29**	43/428 (10.0%)	184/3,365 (5.5%)	4/22 (18.2%)	48/344 (14.0%)
	**30-34**	79/1,057 (7.5%)	203/4,734 (4.3%)	10/59 (16.9%)	63/553 (11.4%)
	**35-39**	80/1,498 (5.3%)	136/4,415 (3.1%)	7/59 (11.9%)	65/540 (12.0%)
	**40-44**	89/1,582 (5.6%)	114/3,394 (3.4%)	13/90 (14.4%)	54/469 (11.5%)
	**45-54**	96/1,917 (5.0%)	90/3,287 (2.7%)		
	**55-64**	26/611 (4.3%)	23/951 (2.4%)		
	**65+**	0/149 (0.0%)	11/211 (5.2%)		

*Restricted to arms A and B only as CHiP data only available for arms A and B

Abbreviations: ART: antiretroviral therapy; PC: Population Cohort.

Supplementary material S12: Members of the HPTN 071 (PopART) trial study team

We are grateful to all members of the HPTN 071 (PopART) Study Team, and to the study participants and their communities, for their contributions to the research.

The HPTN 071 (PopART) Study Team: Richard Hayes (London School of Hygiene & Tropical Medicine, UK), Sarah Fidler (Imperial College, UK), Nulda Beyers (University of Stellenbosch, South Africa), Helen Ayles (Zambart, Zambia; and London School of Hygiene & Tropical Medicine, UK), Peter Bock (University of Stellenbosch, South Africa), Wafaa El-Sadr (HIV Prevention Trials Network [HPTN] Leadership and Operations Centre, USA), Myron Cohen (HPTN Leadership and Operations Centre, USA), Susan Eshleman (HPTN Laboratory Centre, Johns Hopkins University, USA), Yaw Agyei (HPTN Laboratory Centre, Johns Hopkins University, USA), Estelle Piwowar-Manning (HPTN Laboratory Centre, Johns Hopkins University, USA), Virginia Bond (Zambart, Zambia; and London School of Hygiene & Tropical Medicine, UK), Graeme Hoddinott (University of Stellenbosch, South Africa), Deborah Donnell (HPTN Statistical and Data Management Centre [SDMC], USA), Sian Floyd (London School of Hygiene & Tropical Medicine, UK), Ethan Wilson (HPTN SDMC, USA), Lynda Emel (HPTN SDMC, USA), Heather Noble (HPTN SDMC, USA), David Macleod (London School of Hygiene & Tropical Medicine, UK), David Burns (NIAID, USA), Christophe Fraser (University of Oxford, UK), Anne Cori (Imperial College, UK), Nirupama Sista (HPTN Leadership and Operations Centre, USA), Sam Griffith (HPTN Leadership and Operations Centre, USA), Ayana Moore (HPTN Leadership and Operations Centre, USA), Tanette Headen (HPTN Leadership and Operations Centre, USA), Rhonda White (HPTN Leadership and Operations Centre, USA), Eric Miller (HPTN Leadership and Operations Centre, USA), James Hargreaves (London School of Hygiene & Tropical Medicine, UK), Katharina Hauck (Imperial College, UK), Ranjeeta Thomas (Imperial College, UK), Mohammed Limbada (Zambart, Zambia), Justin Bwalya (Zambart, Zambia), Michael Pickles (Imperial College, UK), Kalpana Sabapathy (London School of Hygiene & Tropical Medicine, UK), Ab Schaap (Zambart, Zambia; and London School of Hygiene & Tropical Medicine, UK), Rory Dunbar (University of Stellenbosch, South Africa), Kwame Shanaube (Zambart, Zambia), Blia Yang (University of Stellenbosch, South Africa), Musonda Simwinga (Zambart, Zambia), Peter Smith (Imperial College, UK), Sten Vermund (HPTN Executive Committee), Nomtha Mandla (University of Stellenbosch, South Africa), Nozizwe Makola (University of Stellenbosch, South Africa), Anneen van Deventer (University of Stellenbosch, South Africa), Anelet James (University of Stellenbosch, South Africa), Karen Jennings (City Health Department, Cape Town, South Africa), James Kruger (Department of Health, Western Cape), Mwelwa Phiri (Zambart, Zambia), Barry Kosloff (Zambart, Zambia; and London School of Hygiene & Tropical Medicine, UK), Lawrence Mwenge (Zambart, Zambia), Sarah Kanema (Zambart, Zambia), Rafael Sauter (University of Oxford, UK), William Probert (University of Oxford, UK), Ramya Kumar (Zambart, Zambia; and London School of Hygiene & Tropical Medicine, UK), Ephraim Sakala (Zambart, Zambia), Andrew Silumesi (Ministry of Health, Zambia), Tim Skalland (HPTN SDMC, USA), Krista Yuhas (HPTN SDMC, USA).
